# Chronic kidney disease in the Top End of the Northern Territory of Australia, 2002–2011: a retrospective cohort study using existing laboratory data

**DOI:** 10.1186/s12882-015-0166-6

**Published:** 2015-10-22

**Authors:** Paul D. Lawton, Joan Cunningham, Narelle Hadlow, Yuejen Zhao, Matthew D. Jose

**Affiliations:** Menzies School of Health Research, Charles Darwin University, Darwin, NT Australia; PathWest Laboratory Medicine, Perth, WA Australia; Health Gains Planning Branch, Department of Health, Darwin, NT Australia; University of Tasmania, Hobart, TAS Australia

**Keywords:** Chronic kidney disease, Estimated glomerular filtration rate, Creatinine, Urinary albumin-creatinine ratio, Public health surveillance

## Abstract

**Background:**

The Northern Territory of Australia has a very high incidence of treated end-stage kidney disease (ESKD), largely confined to Indigenous Australians living in remote, under-resourced areas. Surveillance of chronic kidney disease (CKD) is still in its infancy in Australia. We estimate the prevalence and rate of progression of measured CKD across a region using inexpensive readily available laboratory information.

**Methods:**

Using a retrospective de-identified extraction of all records with a serum creatinine or urinary albumin-to-creatinine ratio from the single largest ambulatory pathology provider to the Top End of the Northern Territory of Australia between 1st February 2002 and 31st December 2011, the yearly total and age-specific prevalence of measured microalbuminuria, overt albuminuria and estimated glomerular filtration rate (eGFR) <60 ml/min/1.73 m^2^, and the prevalence of progressive CKD, were calculated.

**Results:**

There was a steady increase in the proportion tested across all health districts in the region, more prominent in non-urban districts. In 2009, the regional adult prevalence of measured microalbuminuria and overt albuminuria was as high as 8.1 %, overt albuminuria alone up to 3.0 % and eGFR < 60 up to 2.3 %. Rates of progressive disease were extremely high, particularly for those with albuminuria (53.1–100 % for those with urinary albumin-creatinine ratio > 300 mg/mmol).

**Conclusions:**

The rates of testing, particularly in districts of high measured prevalence of markers of CKD, are encouraging. However, extremely high rates of progressive CKD are troubling. Further describing the outcomes of CKD in this population would require analysis of linked datasets.

**Electronic supplementary material:**

The online version of this article (doi:10.1186/s12882-015-0166-6) contains supplementary material, which is available to authorized users.

## Background

**Fig. 1:**
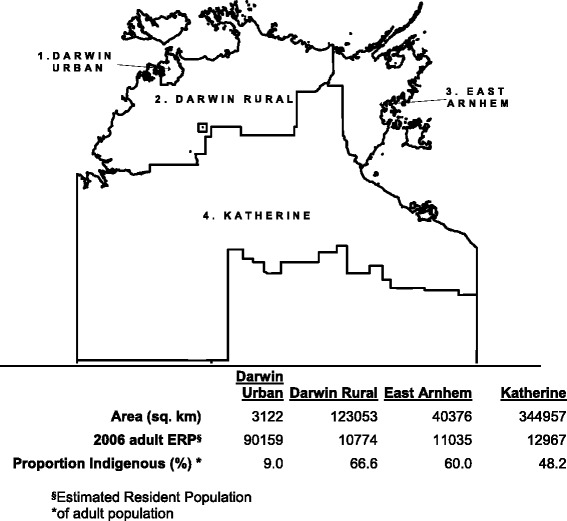
Map of Top End Northern Territory, with 2006 estimated resident population (ERP) by health district including proportion of adult population Indigenous

The Top End of the Northern Territory (NT) of Australia is a large geographical area of over 500,000 square kilometres with a relatively small population comprised of a largely urban centralised non-Indigenous population and a smaller, largely remote, de-centralised and disadvantaged Indigenous population (Fig. 1) that is under-enumerated and culturally and linguistically heterogeneous [1]. The poor health status of its Indigenous population with high rates of chronic kidney disease (CKD) is a public health concern in the NT [2]. There is a single tertiary referral centre staffed by nephrologists in Darwin.

The incidence of treated end-stage kidney disease (ESKD) in the NT is 3–4 times national Australian figures, and is largely confined to the Indigenous community that comprises a third of the NT’s population [3]. At least one individual community has a reported ESKD incidence up to 25 times the national rate [4], amongst the highest in the world. Reports from cross-sectional surveys of a few individual Top End Indigenous communities have shown a very high prevalence of albuminuria [5–8]. However, it is not clear if this means that the high incidence of treated ESKD is a result of a large burden of earlier stages of CKD, a rapid rate of progression or a higher survival rate of those with CKD to end-stage.

The recent validation of the CKD-EPI equation for Indigenous Australians [9], the publication of position papers regarding the use of the CKD-EPI formula for all Australians [10] and the classification and risk stratification of CKD [11] support a population-based approach to determining CKD prevalence.

A few studies have examined clinical laboratory results to determine the prevalence of measured CKD across regions [12, 13], including one in the Australian state of Tasmania [14]. While this method cannot take the place of a population-based random sample, it can be an important adjunct if the population is hard to reach due to remoteness, health service limitations or cross-cultural and linguistic challenges. It can be particularly useful in areas where there is heightened awareness of CKD and strategies to detect and manage it that lead to a substantial proportion of the population at risk to be tested as part of routine clinical care. All of these circumstances apply in the Top End of the NT [2]. For the time of this study the Top End region of the NT was served by one dominant provider of ambulatory pathology services, Western Diagnostic Pathology (WDP, Myaree, Western Australia, a private laboratory providing pathology services across the NT and WA); almost all services for dialysis and transplant patients were provided through an alternative, NT Department of Health hospitals pathology. This allowed calculation of the prevalence of measured CKD that largely excluded those with treated ESKD without the need for data linkage, which has particular ethical and technical challenges for Indigenous Australians [15] and is time consuming, expensive and still in a capacity-building phase in Australia.

The aim of this study was to examine CKD prevalence and rate of progression over a 10 year period in a geographical area with a high incidence and prevalence of treated ESKD using inexpensive readily available information.

## Methods

### Study design and population

A retrospective cohort study was performed using de-identified pathology records with NT postcodes from 1st February 2002 to 31st December 2011 from a single pathology provider. The population included all those with records of a serum creatinine or urinary albumin-to-creatinine ratio (UACR) analysed by WDP.

All records were identifiable only by a laboratory unique identifier for each individual: linkage of records to an individual was performed for clinical purposes at the laboratory level and no other linkage was performed. Records were matched to health districts within the Top End region using the individual’s postcode recorded at the time of testing and 2006 postal area concordance files available from the Australian Bureau of Statistics (ABS) [16] and the NT Health Department’s Health Gains Planning Branch (HGP) [17].

### Laboratory measures

All serum samples were collected into Becton Dickinson serum separator tubes (BD SST II, Becton Dickinson, North Ryde, NSW, Australia). Serum and urine creatinine were measured using the Siemens ADVIA 2400 Jaffe creatinine assay (Siemens Ltd. Australia and New Zealand—Diagnostics Division, Tarrytown, N.Y, USA), an alkaline picrate kinetic method with blank correction. Creatinine was standardised to isotope dilution mass spectrometry (IDMS) standards on 1st February 2002. Percentage coefficients of variation (CVs) within run for quality control material over an indicative 12 month period were: Level 1 (serum creatinine 74.5 umol/L) 1.57–2.1 % CV, Level 2 (serum creatinine 523.9 umol/L) 1.32–6.91 % CV. Urine albumin was measuredusing the Advia Chemistry 2400 method, and percentage CVs between runs were: Level 1 (urine albumin 13.22 mg/L) 5.7 % CV, Level 2 (urine albumin 63.27 mg/L) 2.54 % CV. UACR was reported in mg/mmol.

### Outcome measures

The estimated glomerular filtration rate (eGFR) was calculated using the CKD-EPI equation, as recommended and validated for both Indigenous and non-Indigenous Australians [9, 10]. Mean eGFR was calculated for each year with available data, similar to previous work [14]; mean UACR was calculated similarly. Individuals were then grouped based on their mean eGFR and UACR levels into strata chosen to enable comparison with previous literature. Gender-specific cut-offs were used to define microalbuminuria: for men ≥ 2.5 mg/mmol, for women ≥ 3.5 mg/mmol.

### Statistical analysis

Those with results in years before and after the year of interest, but not in the year of interest, could reasonably be assumed to be still alive in the Top End and were eligible to be counted towards the prevalence numerator for that year. Their result for the year of interest was assumed to be the same as their previous measured result until a new value supplanted it. For example, a person with a mean eGFR in 2002, but no further results until 2005, was assumed to be alive in the Top End and have the same eGFR in 2003 & 2004 until the new mean result in 2005; their 2002 mean eGFR counted for the first 3 years to the numerator of the relevant eGFR category.

Population figures for the region were used for the prevalence denominator, taken from yearly ABS Estimated Resident Population (ERP) figures [18] and mapped to NT health districts using HGP population concordance files [17]. All prevalence was expressed in percentages. The total adult population (15 years or greater) was taken as the denominator to account in part for confounding by indication, in that those having tests performed presumably had a clinical indication for them (and were therefore more likely to have disease than those not tested). In addition, using the total population as the denominator ensured that the prevalence expressed would be more reliable as a minimum estimate. Since all available results from the whole population were used and the population was not assumed to be a random sample, confidence intervals were not calculated.

To examine disease progression, only data from those with two or more serum creatinine measurements at least 2 years apart was used. Progressive CKD was defined as an average annual decline in eGFR during follow-up of ≥2.5 ml/min/1.73 m^2^ per year and a last eGFR value < 45 ml/min/1.73 m^2^, independent of baseline eGFR level [11]. Average annual decline in eGFR was calculated as last available eGFR minus baseline eGFR divided by follow-up time (in years, minimum two) between the two observations [11]. The prevalence of progressive CKD was expressed as a percentage of the tested population.

Ethical approval was given by the combined Human Research Ethics Committee of the Northern Territory Department of Health and Menzies School of Health Research (HREC-2011-1566).

## Results

Those with interstate or overseas postcodes were excluded (15,307 tests), as were those aged <15 years (13,340 tests about 9605 individuals), as the CKD-EPI formula performs poorly in children and adolescents [19]. Those with serum creatinine <25 micromol/L were also excluded as in other studies (249 tests about 146 individuals) [13, 14]. Another 8017 tests about 1829 individuals did not match to a valid postcode, and were excluded. The remaining 495,672 tests about 127,526 individuals were included for analysis.

**Fig. 2:**
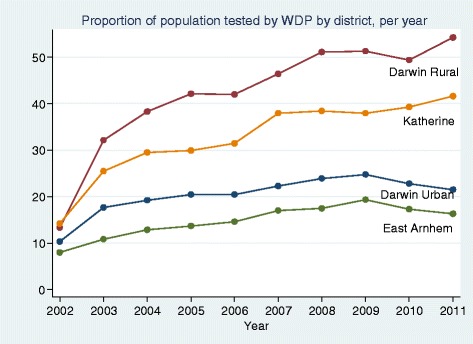
Proportion of adult population tested by Western Diagnostic Pathology by health district, per year

Population characteristics are outlined in Table 1. Over most of the period, there was an increase in the number of people being tested with either a serum creatinine or UACR; this was consistently weighted towards the major population centre, the Darwin Urban district. However, the proportion of the regional population tested was highest in the Darwin Rural district and lowest in the East Arnhem district over the period (Fig. 2). In each region, there was a rise in proportion tested with increasing age for age groups up to 74 years. In every age group 4–6 % more women than men were tested. In any year, most people tested had only one test. For example, in 2009, 95.2 % of those with a serum creatinine and 99.9 % of those with a UACR had only one test.

**Table 1:** Baseline characteristics by year

Year	Individuals tested with eGFR	Individuals tested with UACR	Mean Age	Male (%)	Darwin Urban (%)	Darwin Rural (%)	East Arnhem (%)	Katherine (%)
2002	10,046	1141	47.3	48.3	66.9	7.2	10.4	15.5
2003	18,223	1968	46.7	48.0	65.1	9.7	9.7	15.5
2004	20,126	2813	46.5	49.4	63.6	10.2	10.4	15.8
2005	21,764	3562	46.1	47.7	64.1	10.6	10.5	14.7
2006	23,495	3128	46.8	47.6	64.2	10.4	10.6	14.8
2007	27,347	3231	46.4	47.7	63.1	10.3	10.7	15.9
2008	29,709	3942	46.0	48.2	63.6	10.5	10.7	15.2
2009	31,788	3881	46.2	47.2	64.3	10.3	11.0	14.5
2010	30,507	3535	46.4	47.1	62.8	10.6	10.8	15.8
2011	30,246	3616	45.6	47.0	60.3	11.8	11.1	16.8

A sharp rise in absolute numbers tested (Table 1) and in the proportion of the total population tested was noted from 2002 to 2004, with subsequent smaller yearly rises to 2008–9 and then a plateau, with similar patterns in each region (Fig. 2) and for both serum creatinine and UACR tests (data not shown).

**Fig. 3:**
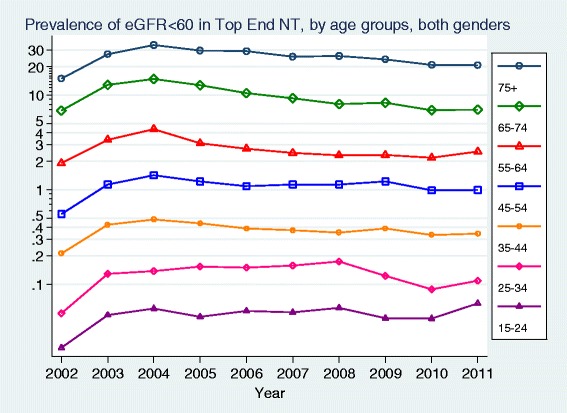
Prevalence of eGFR < 60 ml/min/1.73 m2 in Top End NT, by age groups, both genders

The prevalence of measured moderate to severe CKD (eGFR < 60 ml/min/1.73 m^2^) increased markedly with age (Fig. 3). Overall the prevalence (up to 2.3 %) was 1.5–2 times higher in health districts outside Darwin Urban (Table 2).

**Table 2:** Prevalence (%) of Markers of CKD by District, 2009^a^

District	Number UACR tests	Percent ERP^b^ tested with UACR	Percent with Mean UACR ≥ 2.5/3.5	Percent with Mean UACR ≥ 30	Number eGFR tests	Percent ERP^b^ tested with eGFR	Percent with Mean eGFR < 60
Darwin Urban	7070	7.2	1.6	0.5	28,790	29.2	1.1
Darwin Rural	2295	19.5	8.1	2.8	4147	35.2	1.8
East Arnhem	2223	18.5	6.9	2.4	4458	37.1	1.5
Katherine	2974	20.9	8.1	3.0	6132	43.2	2.3

^a^ Represents 2009 results as well as results prior to 2009 carried forward if there were no 2009 results but both pre-2009 and post-2009 data were available (see Methods)

^b^ Estimated Resident Population 15 years and over

**Fig. 4:**
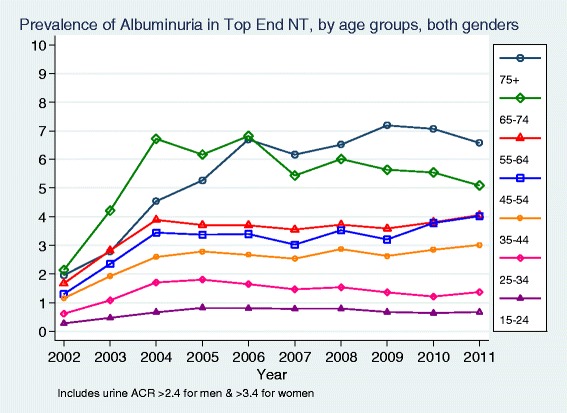
Prevalence of Albuminuria in Top End NT, by age groups, both genders

In general, the prevalence of measured microalbuminuria and overt albuminuria (Fig. 4) increased with age after 2004; the considerable volatility in older age groups was consistent with relatively small absolute numbers. Overall the prevalence of those with a UACR ≥ 2.5/3.5 (up to 8.1 %) or a UACR ≥ 30 (up to 3.0 %) was four to six times higher in districts outside Darwin Urban (Table 2).

Over the whole study period, 39,850 people had ≥2 serum creatinine levels separated by at least 2 years; median follow-up was 6.8 years with a maximum of 9.9 years. Of these, 1159 people (2.9 % of those assessed) met the definition for progressive CKD. Table 3 outlines the risk of progression according to initial CKD stage, and shows the importance of the initial degree of albuminuria and (to a lesser extent) level of eGFR to the risk of progression.

**Table 3:** Percentage^a^ with progressive CKD^b^, whole time period

Initial eGFR group	Initial UACR <3.4	Initial UACR 3.4–9.9	Initial UACR 10–29	Initial UACR 30–299	Initial UACR >300	Missing UACR
105+	0.3	1.9	1.7	7.9	53.1	0.2
90–104	0.6	2.5	3.7	13.9	66.6	0.3
75–89	1.7	3.0	4.3	23.0	61.7	1.3
60–74	3.6	15.4	22.5	40.1	71.8	5.7
45–59	6.4	10.6	20.9	42.2	95.6	13.4
30–44	4.9	7.8	25.6	57.6	84.3	26.1
15–29	0.0	11.0	19.2	56.2	55.0	34.9
0–14	0.0	0.0	0.0	13.8	100.0	9.9

^a^ Percentage is of those with 2 or more creatinine measurements at least 2 years apart (*N* = 39,850)

^b^ Progressive CKD defined as average annual decline of >2.5 ml/min/1.73 m^2^/year, with final eGFR <45 ml/min/1.73 m^2^, minimum follow-up 2 years [11]

## Discussion

This study describes the overall prevalence of measured CKD in a region with very high incidence of treated ESKD and a single dominant (albeit not exclusive) pathology service provider. Previous cross-sectional studies in the region (Additional file 1: Table S1) have described a very high prevalence of markers of kidney disease in individual remote communities largely populated by Indigenous Australians [3–5] and a somewhat lower prevalence amongst Indigenous people of the Darwin Urban region [8]. This study, using existing clinical pathology data rather than community-wide screening or a population-based random or weighted sample, has demonstrated a still lower prevalence of CKD, although this is likely to represent a minimum figure that is still higher than national estimates.

This lower prevalence could be explained partly by the “whole of population” approach, which includes a large number of non-Indigenous people at lower risk of kidney disease. The CKD-EPI formula used in this study will more correctly estimate a lower proportion with moderate to severe CKD than the MDRD formula used in previous studies [9, 20]. In addition, the relatively high proportion missing UACR tests underestimates the tabulated prevalence of those with CKD and higher (that is, normal or “near-normal”) eGFRs. These individuals appear to behave more like those with normal or “near-normal” UACRs when progression is examined.

This is the first study to demonstrate the rate of CKD progression in the region, which appears to be much higher than that demonstrated in “high-risk population” studies elsewhere [21]. The degree of proteinuria is a potent predictor of CKD progression, as widely described elsewhere [21, 22].

There was an initial and substantial rise in the numbers tested and in the measured prevalence of CKD markers from 2002 to 2004. This is well beyond that expected from an initial incomplete year (2002 results only are from 1st February due to the commencement of IDMS standardisation). Although there were changes in reimbursement for health checks for Indigenous adults (introduced in 1999 for those over 55, and 2004 for those aged 15–55 years), these changes are unlikely to be the main reason for the increase because of the low uptake of these services [23]. Rather, it is probably best explained by increased testing as a result of the implementation of changes in non-communicable chronic disease care in the Top End in 2002, including increased funding and resources from changes to pharmaceutical funding (fully reimbursing costs of medications used in remote communities) [24] and the commencement and expansion of remote primary-care based chronic disease quality improvement research [25].

There was a subsequent steady fall in the prevalence of measured moderate to severe CKD (eGFR < 60 ml/min/1.73 m^2^) as the population size grew and the proportion of the population tested slowly increased, despite the prevalence of albuminuria increasing. This suggests that those with more severe kidney disease were identified by health services early in the time period studied. It also suggests that either there are many with earlier stage CKD (particularly with albuminuria alone) who are still to be identified, or alternatively that in aggregate across the region the prevalence of markers of earlier stage CKD is lower than the individual community data previously published.

There were large differences between the age-specific prevalence rates in this study and those reported in the recently released Australian National Health Measures Survey (NHMS), although such comparisons must be made with extreme caution as remote areas were excluded from the NHMS and the proportion of Indigenous respondents was very small [26]. Whilst the prevalence of moderate to severe CKD (eGFR < 60 ml/min/1.73 m^2^) in those over 65 years was similar in this study and in the NHMS, rates for those between 35 and 65 years in the Top End of the NT were double national rates. In contrast rates of albuminuria in the Top End were significantly lower than national rates at every age group. This also suggests either that there are many more people with albuminuria yet to be identified in the Top End of the NT or that they were tested using point-of-care technology rather than laboratory testing [27].

Also relevant are comparisons with data from the recently released National Aboriginal and Torres Strait Islander Health Measures Survey (NATSIHMS), which aimed to be a representative sample of Indigenous Australians from 2012–13 [28]. The prevalence of those with eGFR < 60 ml/min/1.73 m^2^ in non-urban health districts was slightly lower in these Top End data than in the NATSIHMS; again, rates of albuminuria in these Top End data were significantly lower than national Indigenous rates. Unfortunately, NATSIHMS rates by age groups have not been released for comparison.

To our knowledge, this is the first study to use methodology that includes cases known to be alive, despite not being tested, in interval years to calculate prevalence. This “carrying forward” approach may underestimate fluctuations in kidney function and albuminuria over time. This approach also assumes that the Top End’s population does not leave and then return to the Top End after long periods of time. Whilst an estimate of those returning to the NT is not available, 2001 ABS census data estimated that 89.4 % of Indigenous Top End residents and 67.0 % of non-Indigenous Top End residents lived in the same Health District 5 years before [29].

In the vast majority of individuals, calculation of mean eGFR and mean UACR relied upon one test only, and this might result in an overestimate in prevalence of CKD markers, particularly low-level albuminuria [30, 31]. The collection of these data predated revisions to the definition of CKD that now incorporate both eGFR and UACR simultaneously; during the time of this study testing was sequential (based on local guidelines) and eGFR-UACR “paired” samples uncommon.

Data about the proportion of tests processed by individual pathology providers in Australia are closely held by government and thought “commercially sensitive”; as a result, it is not possible to document the extent of Western Diagnostic Pathology’s dominance in the region over the time of study. Replicating this work for the whole of the NT or for other Australia states would require linking records from more than one pathology provider. Because Indigenous status is not recorded in pathology data, calculating separate Indigenous and non-Indigenous estimates of CKD prevalence would require linkage with other datasets that include an Indigenous identifier (such as hospitalisation data). Linkage would also reduce the chances of multiple identifiers leading to an overestimate of prevalence, particularly for Indigenous Territorians.

The absence of dataset linkage also means that this study is unable to determine accurately the total number of ESKD cases not receiving treatment, as it is possible that some of those receiving RRT had some blood tests through Western Diagnostic Pathology even though the dominant pathology provider for these individuals would have been the public hospital based pathology service.

This study used existing clinical pathology data rather than a population-based random sample or community-wide screening as attempted in other studies summarised in the Additional file 1: Table S1. As a result, inferences drawn about those not tested, and the population as a whole, are limited. Different approaches to testing for CKD in urban and remote areas of the Top End of the NT are likely to limit the validity of comparisons between them, given specific guidelines [32] and awareness of the heightened ESKD risk for Indigenous people in remote areas of the NT. The prevalence figures shown, however, do provide minimum estimates for the whole population; they are useful in the absence of a population based random sample of the region which is unlikely in the foreseeable future due to the competing demands and prohibitive expense of research in such a remote, culturally diverse environment.

The prevalence of measured CKD, even if the whole population were tested, is only one measure of the burden of disease. Because prevalence is related to both incidence and duration, substantial differences in the incidence of and survival from disease may be masked within similar prevalence figures between populations.

## Conclusions

This study provides useful information for planning and policy development. Both the rate of testing and the minimum estimates of the prevalence of markers of CKD are much higher in non-urban health districts of the Top End, but still lower than previous community-based surveys. Rates of moderate to severe CKD in middle age in the Top End are double national figures. As testing rates have increased over the last decade, the prevalence of measured albuminuria has increased but moderate to severe CKD has not. The rate of CKD progression is much higher than that published for other high-risk populations. To determine better the incidence and outcomes of CKD across this or other regions, including its association with Indigenous status, studies using linked data from multiple sources will be required. Now that data linkage systems are developing in Australia, it should be possible to do this in the near future.

## Additional file

Additional file 1: Table S1. Comparison of overall prevalence of markers of CKD in different studies in Top End NT region & nationally (DOCX 15 kb)

## Abbreviations

NT: Northern Territory (of Australia)
CKD: Chronic kidney disease
ESKD: End-stage kidney disease
ABS: Australian Bureau of Statistics
HGP: Health Gains Planning Branch (of the Northern Territory Department of Health)
eGFR: Estimated glomerular filtration rate
CKD-EPI: Chronic kidney disease epidemiology collaboration
CV: Coefficient of variation
ERP: Estimated resident population
UACR: Urine albumin-to-creatinine ratio
NHMS: National Health Measures Survey
NATSIHMS: National Aboriginal and Torres Strait Islander Health Measures Survey
MDRD: Modification of diet in renal disease
