# Supplementary material for: Chronic kidney disease in the Top End of the Northern Territory of Australia, 2002–2011: a retrospective cohort study using existing laboratory data
Source: BMC Nephrol. 2015 Oct 22;16:168. doi: 10.1186/s12882-015-0166-6 (PMC4619033; doi:10.1186/s12882-015-0166-6)
Supplement: Additional file 1: Table S1. — Comparison of overall prevalence of markers of CKD in different studies in Top End NT region & nationally (DOCX 15 kb) [file 12882_2015_166_MOESM1_ESM.docx]

**Additional file 1: Table S1. Comparison of overall prevalence of markers of CKD in different studies in Top End NT region & nationally**

| **Study** | **Number tested** | **Percent of eligible population tested** | **Ages** | **Microalbuminuria (%)** | **Macroalbuminuria (%)** | **eGFR<60 (%)** | **eGFR method** | **Ethnicity** | **Location** | **Sample** |
| --- | --- | --- | --- | --- | --- | --- | --- | --- | --- | --- |
| **Hoy et. al., 2001** | 956 | 76.2 | 20+ | 24 | 18.9 | 6 | MDRD-186 | Indigenous only | NT Remote | Community screening |
| **McDonald et. al., 2003** | 237 | 58 | 18+ | 31 | 13 | N/A | MDRD-186 | Indigenous only | NT Remote | Community screening |
| **Shemesh et. al., 2007** | 379 | 53.7 | 15+ | 23.7 | N/A | N/A | N/A | Indigenous only | NT Remote | Community screening |
| **Maple-Brown et. al., 2011** | 860 | 14 | 15+ | 10.5 | 4.3 | 2.4 | MDRD-186 | Indigenous only | NT Urban | Regional volunteer survey |
| **White et. al., 2010 (AusDiab)** | 11247 | 55.3 | 25+ | 6.3 | 1.1 | 3.4 | CKD-EPI | Mostly non-Indigenous | Australia Mostly urban | Nationally representative weighted survey |
| **NHMS/AHS, 2013** | 11248 | 37.1 | 12+ | 6.7 | 0.9 | 3.6 | CKD-EPI | Mostly non-Indigenous | Australia Mostly urban | Nationally representative weighted survey |
| **NATSIHMS, 2014** | 8157 | 40.4 | 18+ | 12.5 | 4.1 | 2.5 | CKD-EPI | Indigenous only | Australia Urban & Remote | Nationally representative weighted survey |
